# Research Progress on the Preparation and High-Value Utilization of Lignin Nanoparticles

**DOI:** 10.3390/ijms23137254

**Published:** 2022-06-29

**Authors:** Kefeng Liu, Yuntang Zhuang, Jiachuan Chen, Guihua Yang, Lin Dai

**Affiliations:** 1 Key Laboratory of Pulp and Paper Science & Technology of Ministry of Education, Qilu University of Technology (Shandong Academy of Sciences), Jinan 250353, China; kfliu@qlu.edu.cn; 2 State Key Laboratory of Biobased Material and Green Papermaking, Qilu University of Technology (Shandong Academy of Sciences), Jinan 250353, China; yuntang1110@163.com (Y.Z.); chenjc@qlu.edu.cn (J.C.); 3 College of Light Industry and Engineering, Tianjin University of Science and Technology, Tianjin 300457, China

**Keywords:** lignin, nanoparticles, preparation methods, high-value utilization

## Abstract

Lignin nanoparticles, the innovative achievements in the development and utilization of lignin, combine the structural characteristics of nanomaterials and lignin molecules and have a wide range of applications. In this review, we summarize the methods for preparing lignin nanoparticles by solvent exchange method, mechanical method, biological enzymatic method, interface polymerization/crosslinking method, and spray freezing method, and emphatically introduce the application prospects of lignin nanoparticles in ultraviolet protection, antibacterial, nano-filler, drug delivery, and adsorption, aiming to provide a certain reference direction for additional high-value applications of lignin nanoparticles.

## 1. Introduction

In recent years, with the excessive consumption of fossil energy and environmental pollution becoming more and more serious, natural biomass materials have been widely studied by researchers due to their advantages of wide distribution, abundant resources, low pollution, and renewability [1]. The efficient development and utilization of natural biomass materials plays a very positive role in solving the problem of waste biomass resources and low utilization rate in environmental protection, medical treatment, the chemical industry, and other fields. Lignin, the second largest biomass resource after cellulose in nature, is the most abundant renewable aromatic organic compound discovered so far [2]. However, although more than 50 million tons of lignin are extracted in the pulp and paper industry every year, only about 5% of lignin can be utilized efficiently, and most of it is directly discharged or burned as a by-product of the paper industry, which not only causes a great waste of resources, but also pollutes the environment [3].

**Figure 1:**
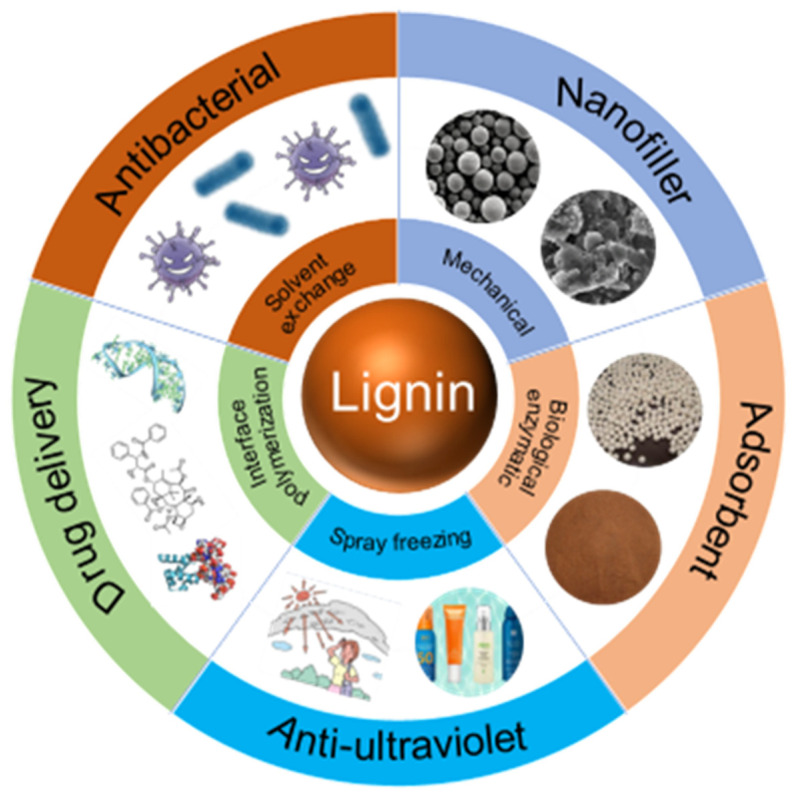
Preparation methods and application of lignin nanoparticles.

With the rapid development of modern nanotechnology, the generation of lignin nanoparticles (LNPs) provides a new direction for the high-value utilization of lignin [4]. Researchers have extensively studied the application fields of LNPs. Lignin colloidal spheres [5] and lignin-based composite nanoparticles [6] have the advantages of UV resistance, antibacterial properties, and non-cytotoxicity [7,8], showing great application potential as natural sunscreen, drug carriers, and nano filler, etc. (Figure 1).

This review summarizes the preparation methods and application status of LNPs, hoping to provide reference for the in-depth research of lignin processing technology, and the research and development of LNPs related products.

## 2. Preparation of Lignin Nanoparticles

### 2.1. Solvent Exchange Method

The preparation of LNPs by solvent exchange is a typical method, the process is as follows: lignin (hydrophobic group) in organic solvent and non-solvent (usually water) form precipitate under certain conditions, and the precipitate is washed and dried to prepare corresponding LNPs. The solvent exchange method has the advantages of simple operation, mild conditions, and adjustable particle size of products [9]. Common precipitation methods include the self-assembly method, dialysis method, acid precipitation method, etc.

#### 2.1.1. Self-Assembly Method

The self-assembly method is a method which connects atoms, ions, and molecules together to construct a nanoscale structure through the action of non-covalent bonding forces between molecules. The advantages of this method include its simple process and stable and controllable size, making it a common method for preparing LNPs [10].

Li et al. prepared nanospheres by self-assembly using waste bamboo chips as raw material after acetic acid extraction [11]. Taking advantage of the high solubility and high phenolic hydroxyl content of the low-molecular-weight lignin obtained by fractionation, it is self-assembled to synthesize nanospheres with uniform morphology and size. The result shows that the nanospheres prepared by self-assembly under ultrasound are more dispersed, smoother, and more uniform in size than those prepared by direct magnetic stirring. Compared with the original lignin, the nanospheres have fluffier configuration, higher specific surface area, and show shallow coloration.

Wang et al. modified lignin by microwave acetylation, which used acetic anhydride as the reaction reagent and dispersion solvent, and prepared high-yield LNPs by solvent transfer combined ultrasonic method [12]. The prepared LNPs can be formed quickly without dialysis and can be easily separated by centrifugation. At the same time, the THF used in the experiment can be recycled, which is beneficial to reducing the cost, simplifying the process, and achieving large-scale industrial production. It is found that the maximum yield of LNPs could reach 82.3% with the increase of initial lignin concentration and ultrasonic intensity.

Xiong et al. prepared single-pore lignin hollow nanospheres by direct self-assembly method [13]. The enzymatic hydrolysis lignin (EHL) was dissolved in THF and then deionized water was gradually dropped into the lignin/THF mixture to form lignin hollow nanospheres. The dispersion of nanoparticles is stable when the pH is between 3.5 and 12. As the initial lignin concentration increases, the diameter of the nanoparticles and the thickness of the shell wall increased, while the single pore diameter, specific surface area, and pore volume decreased.

**Figure 2:**
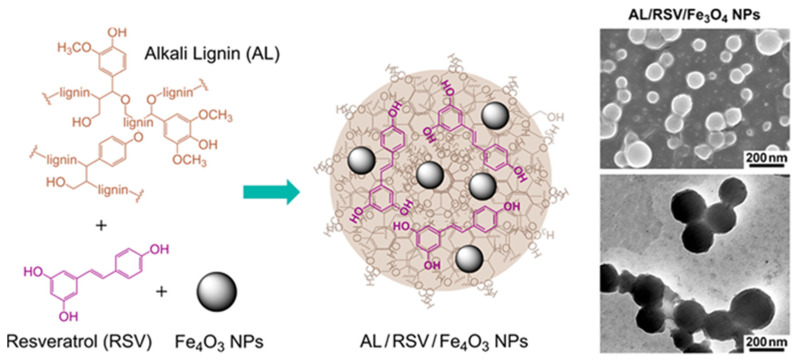
The preparation process, SEM, and TEM images of AL/RSV/Fe_3_O_4_. Reprinted with permission from Ref. [14]. Copyright 2017 American Chemical Society.

Dai et al. prepared spherical nanoparticles with good dispersion by a simple self-assembly method, using alkali lignin (AL) as raw material and methanol solution as dispersant [14]. The study found that when the initial concentration of alkali lignin in methanol is 0.5 mg/mL and the final water content is 90%, the particle size of lignin nanoparticles is about 130 nm and the dispersion is uniform (Figure 2). The self-assembly of AL with bioactive molecules resveratrol and magnetic nanoparticles can form stable nanodrug carriers. Compared with free drugs, AL/RSV/Fe_3_O_4_ greatly improved the stability of the anti-tumor effect of resveratrol.

Lintinen et al. synthesized colloidal lignin particles (CLPs) by dissolving lignin in a mixture of THF and ethanol and subsequently introducing the lignin solution into water. The organic solvent of this method can be recycled and reused by rotary evaporator, laying the foundation for the recycling production of CLPs [15].

Li et al. studied a direct self-assembly method for the formation of kraft lignin (KL) nanocapsules in a mixed solvent of ethanol and water [16]. Among them, π–π interaction between aromatic rings plays an important driving role in the formation of KL nanocapsules. The size of the prepared KL nanocapsule can be controlled in the range of tens to hundreds of nanometers, which is suitable for different fields.

**Figure 3:**
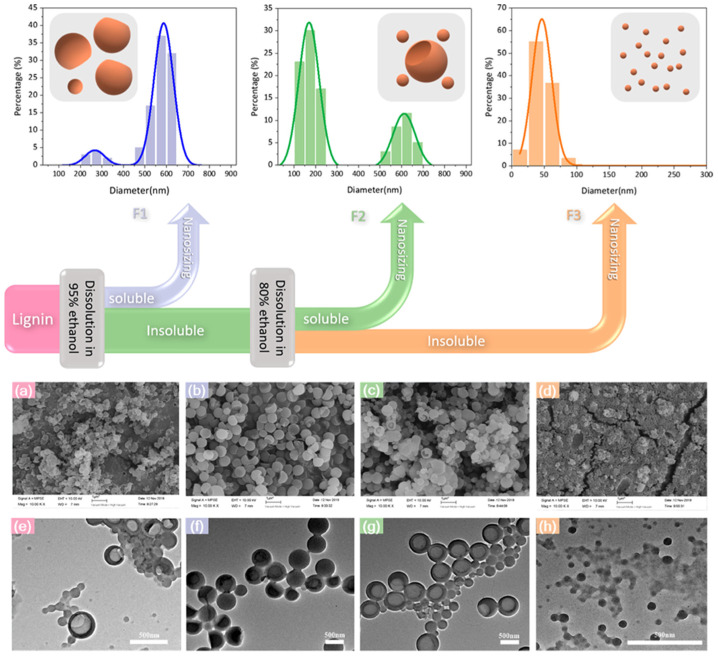
Lignin fractionation is proposed as an efficient way to reduce heterogeneity in lignin self-assembly nanosizing and produce uniform lignin nanoparticles with small size; SEM images of LMNPs prepared from EHL (**a**), F1 (**b**), F2 (**c**), and F3 (**d**); TEM images of LMNPs prepared from EHL (**e**), F1 (**f**), F2 (**g**), and F3 (**h**). Reprinted with permission from Ref. [17]. Copyright 2020 American Chemical Society.

In order to investigate the effect of heterogeneity of lignin on self-assembled nanoparticles, Pang et al. prepared different lignin micro/nano-particles (LMNPs) by using three lignin fractions (F1, F2, F3) separated by enzymatic hydrolysis of lignin as raw materials [17]. The LMNPs prepared by the three components show different morphological characteristics: large and incomplete spherical particles with a diameter of 450–650 nm were synthesized from F1; F2 was used as the raw material to prepare two different morphological particles, one with a hollow structure of large size (500–700 nm) and the other with a dense structure of small size (100–250 nm); using F3 as the raw material, small size particles (about 50 nm) with uniform particle size distribution and compact structure were formed (Figure 3). It is worth noting that the LNPs prepared by F3 have the advantages of high yield, small particle size, uniform distribution, and high stability of water dispersion. Through the analysis of the formation mechanism of the three particles, the authors found that the inhomogeneity of lignin is the key factor for the different sizes of self-assembled nanoparticles, and the high molecular weight of fractionated lignin has a significant effect on improving the performance of nanoparticles.

#### 2.1.2. Dialysis Method

Lievonen et al. proposed a simple and direct method to prepare lignin nanoparticles [18]. This method can produce spherical LNPs with colloidal stability without chemical modification of lignin. The sulfate lignin was dissolved in THF and dialyzed in deionized water to obtain spherical LNPs with an average particle size of 200–500 nm. The results show that when the pH is between 4 and 10, and the ionic strength is up to 500 mM, the dispersion of LNPs is very stable and favorable for industrial application.

Ma et al. prepared LNPs with controllable particle size by combining the conventional fractionation technique with dialysis [19]. The diameters of the prepared LNPs were in the range of 21–139 nm, where 21 nm is one of the smallest particle sizes reported so far. This method provides a new idea for the production of small-sized LNPs.

Lu et al. proposed a method to prepare LNPs directly from corn straw [20]. They used THF as an organic solvent to separate lignin from corn straw, then placed the obtained lignin solution in a dialysis bag and soaked it in deionized water for 12 h. The LNPs prepared by this method have a smooth surface, controllable size and structure, and good UV resistance properties.

#### 2.1.3. Acid Precipitation Method

Acid precipitation method is to dissolve lignin in organic solvent or alkaline aqueous solution, by reducing the pH value of the solution, so that the lignin molecules in the form of nanoparticle precipitation. This method is one of the most important methods in preparation of LNPs because of its simple operation and low requirements on experimental environment. However, this method requires modification of the lignin to improve its solubility, so as to better control the morphology and size of lignin nanoparticles.

Yang et al. dissolved alkali lignin in ethylene glycol and then adjusted the pH of the solution with different acids (HCl, H_2_SO_4_, and H_3_PO_4_) to prepare LNPs [21]. It was shown that the physical properties of LNPs were significantly affected by the pH of the solution. The size of the prepared LNPs was uniform and varied with the change of pH, which were 32.8 ± 6.0 nm (HCl, pH 2.5), 58.9 ± 8.6 nm (H_2_SO_4_, pH 2.9) and 54.1 ± 6.7 nm (H_3_PO_4_, pH 2.6), respectively. The highest LNPs yield of 87.9% was obtained when pH was 2.5.

Azimvand et al. prepared LNPs by dissolving alkali lignin in polyethylene glycol and adjusting the pH of the solution to 4 using hydrochloric acid [22]. The results show that the diameter of LNPs is suitable when the pH value is 4–6. The smallest particle size of LNPs was in the range of 40–60 nm at pH 4. In addition, the method is easy to operate and the process is green, which is suitable for further large-scale synthesis of LNPs.

**Figure 4:**
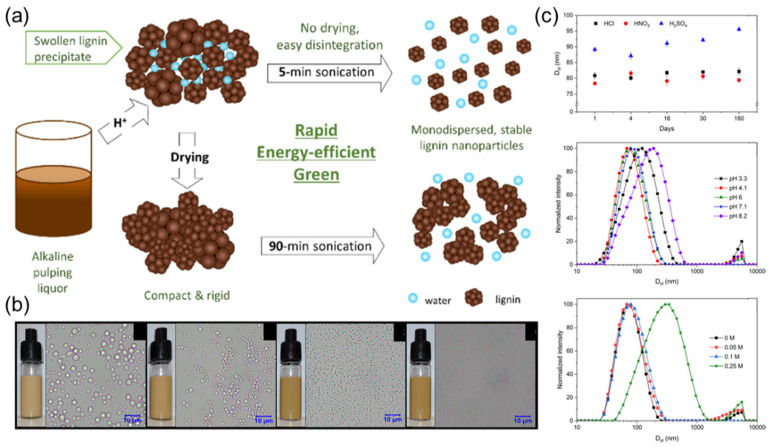
(**a**) A green and rapid method of preparing LNPs directly from a sulfur-free alkaline pulping liquor by combining acid precipitation and ultrasonication; (**b**) the optical images of LNPs with various hydrochloric acid; (**c**) stability of the LNPs monitored by the changes in the average hydrodynamic diameter. Reprinted with permission from Ref. [23]. Copyright 2019 American Chemical Society.

Agustin et al. directly prepared LNPs from alkaline pulping liquor by combining acid precipitation with ultrasonic method [23]. It was found that the yield of LNPs prepared by HCl was the highest, and the diameter of LNPs prepared by three acids (HCl, H_2_SO_4_, and HNO_3_) was less than 100 nm. The DH of LNPs prepared from HCl and HNO_3_ remained constant for 180 days (Figure 4). Furthermore, the LNPs exhibited good emulsification properties, and they could form stable emulsions (oil-in-water) without the addition of surfactants. This process is efficient and green, providing a sustainable development path for solvent-free LNPs production, and has a wide application prospect in the food, medical, and other fields.

Alipoor et al. prepared functionalized LNPs from carboxymethylated or carboxypentylated lignin using an acid deposition method [24]. Firstly, they carboxymethylated lignin with sodium chloroacetate (SCA) and reacted activated lignin with chlorohexanoic acid (CHA) to prepare carboxypentylated lignin. Next, the carboxymethylated or carboxypentylated lignin was mixed with ultrapure water, and the lignin was completely dissolved with sodium hydroxide, and then the pH was adjusted with hydrochloric acid to precipitate LNPs. Both carboxypentylated lignin nanoparticles (PLNPs) and carboxymethylated lignin nanoparticles (CLNPs) prepared by this method have certain adsorption properties, among which the adsorption capacity of PLNPs is larger. This study can achieve precise control of nanoparticle size and regulate the adsorption behavior of LNPs, providing a new idea for the application of lignin nanoparticles in drug delivery and other applications.

The above three solvent exchange methods are often used to prepare LNPs, but there are some disadvantages. For example, lignin cannot be completely uniformly dispersed in the solvent, and agglomeration may occur in some cases. In the subsequent process, the solvent has to be removed by rotary evaporation or freeze-drying, which is unfavorable for precise control of the size and morphology of the nanoparticles.

### 2.2. Mechanical Method

The mechanical method is a preparation method that uses external mechanical shear force or high-frequency vibration to create the lignin interface reaction, so that large particles become small particles [25]. At present, the mechanical method mainly includes high shear homogenization method, ultrasonic method, and so on.

#### 2.2.1. High Shear Homogenization Method

Nair et al. proposed a method to prepare lignin nanoparticles using a high shear homogenizer [26]. It is found that the lignin sulfate particles are completely homogenized and the particle size is less than 100 nm after mechanical shearing for 4 h. Moreover, ^13^C-NMR and ^31^P-NMR spectra analysis showed that after 4 h of mechanical treatment, the chemical composition and structure of lignin nanoparticle did not change significantly compared with the starting sulfate lignin particles.

#### 2.2.2. Ultrasonic Method

Gonzalez et al. prepared LNPs by ultrasonic treatment of softwood lignin, and obtained a lignin/water dispersion system with good colloidal stability [27]. After 6 h of ultrasonic treatment, the particle size of lignin decreased significantly, which could be controlled between 10 and 50 nm. In addition, ultrasonic treatment has a certain chemical modification effect on lignin. Compared with untreated lignin, the polarity of LNPs is enhanced and their stability in water is dramatically improved.

Gilca et al. proposed a physical method that modifies lignin with ultrasound to obtain nanoparticles [28]. Using industrial lignin as raw material, they sonicated the lignin suspension for 60 min to obtain a uniform and stable nano-dispersion system. The anterior and posterior structures were characterized by FTIR-spectroscopy, GPC-chromatography HSQC and ^31^P-NMR-spectroscopy, and the results showed that the composition and structural changes of LNPs were related to the properties of lignin, not to the strength of lignin.

Compared with the solvent exchange method, the mechanical method has the advantages of low cost and environmental protection because it avoids the use of organic solvents. Moreover, mechanical method has the advantages of simple preparation, large-scale application, etc., greatly promotes the application of LNPs in food industry, medicine and other fields. However, the structure and dimensional stability of LNPs prepared by this method are poor.

### 2.3. Enzymatic Hydrolysis

Juikar et al. extracted LNPs from coconut fibers by microbial hydrolysis and compared them with LNPs prepared by high shear homogenization and ultrasonic methods [29]. The FEG–SEM micrographs of LNPs prepared by homogenization and ultrasonic method showed that the particles aggregated, while the FEG–SEM micrographs of LNPs prepared by microbial method showed that the particles dispersed well. The technology is an efficient and environmentally friendly method for preparing lignin nanoparticles and is also suitable for lignin obtained from other sources such as wood and agricultural biomass.

Rangan et al. prepared lignin-rich nanoparticles using lignocellulose from loofah as the raw material and specific enzymes to decompose lignin-cellulose complex [30]. These nanoparticles were characterized by electron microscopy. The observation results showed that the lignin nanoparticles were cubic with uniform particle size of about 20~100 nm. The nanoparticles with high content of lignin have a wide application prospect in the automobile, pharmaceutical, and other fields.

Juikar et al. used aspergillus oryzae to hydrolyze the bulk lignin in cotton stalks for producing LNPs [31]. Subsequently, they compared it with the lignin nanoparticle produced by high shear homogenization and ultrasonic methods. The results showed that the yield of LNPs produced by the enzymatic hydrolysis method (45.3%) was lower than that of the high shear homogenization method (79.5%) and the ultrasonic method (62.6%). In addition, the prepared LNPs has various properties such as antibacterial, UV protection, and antioxidant, after coating on the surface of cotton and linen fabrics.

The preparation of LNPs by enzymatic hydrolysis methods has the characteristics of mild reaction conditions, simple operation, cleanliness, and efficiency. However, the price of this method is high and the product yield is relatively low.

### 2.4. Interfacial Polymerization/Crosslinking

Yiamsa et al. selected toluene diisocyanate (TDI) to react with the hydroxyl group of lignin to generate a hollow lignin nanocapsule with a hydrophilic nucleus [32]. Firstly, lignosulfonate was dissolved in ultra-pure water to form a dispersed phase, and mixed with cyclohexane containing biocompatible surfactant polyglycerol polyricinoleate (PGPR). Then, the pre-emulsion was stirred at room temperature and treated by ultrasound to obtain stable microemulsion. The solution of TDI in cyclohexane was added to the nano-droplet interface of the microemulsion, and the polymerization reaction was initiated to generate hollow lignin nanocapsules. The wall thickness of the dried capsule is 10~20 nm, and the particle size ranges from 150~200 nm. The capsules can be lysed by laccase and effectively encapsulate bioactive drugs. It is a potential nano container for agricultural applications.

Nypelo et al. proposed a new method to assemble lignin macromolecules into colloidal structures by taking advantage of their aromatic and cross-linking properties [33]. In this study, the authors controlled the size and integrity of the resulting particles by the concentration of surfactants and crosslinking agents. The water-in-oil (W/O) microemulsion was composed of a colloidal dispersion system of non-ionic surfactant and low molecular weight alkali lignin. After self-emulsification, the internal phases rich in lignin were crosslinked to form spherical particles with particle size ranging from 90 nm to 1 mm. Furthermore, the LNPs were found to be effective carriers of Ag nanoparticle.

**Figure 5:**
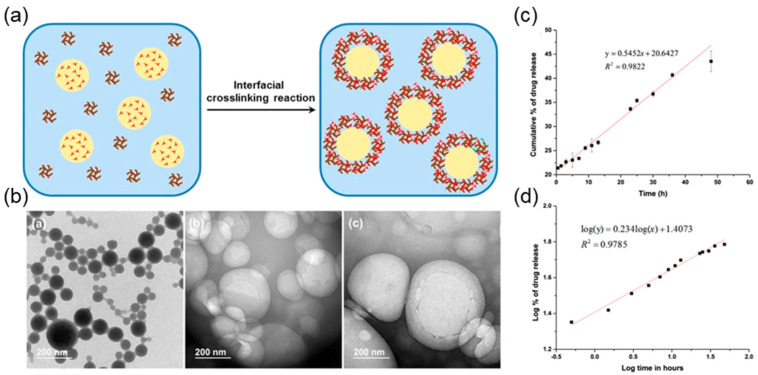
(**a**) Preparation of lignin-based nanocapsules via interfacial miniemulsion crosslinking reaction; (**b**) TEM images of solid particles using styrene as the oil phase in miniemulsion and lignin nanocapsules using butyl acetate as the oil phase in miniemulsion; mathematic models for coumarin-6 release: (**c**) fitting curve of zero-order release at pH 7.4; (**d**) fitting curve of Korsmeyer−Peppas model at pH 4.0. Reprinted with permission from Ref. [34]. Copyright 2016 American Chemical Society.

Chen et al. successfully synthesized lignin-based pH-responsive nanocapsules using lignosulfonate as the starting material through interfacial microemulsion crosslinking method [34]. Firstly, lignin was grafted onto allyl group by etherification, and then ultrasonic dispersed into oil-in-water (O/W) microemulsion system (Figure 5). At the interface of the microemulsion droplet, allyl functionalized lignin and mercaptan crosslinking agent reacted with sulfhydryl radical to form nanocapsules. The particle size of the synthesized lignin nanocapsules is about 100–400 nm. The particle size of the synthesized lignin nanocapsules can be well adjusted by controlling the loading levels of surfactants and stabilizers. It is found that lignin nanocapsules can effectively encapsulate hydrophobic molecules through microemulsion, which has great application potential in the controllable transport of hydrophobic molecules such as drugs, essential oils, and antioxidants.

The interfacial polymerization/crosslinking method for LNPs preparation has many advantages, such as: (1) the particle size is uniform and controllable; (2) LNPs with special properties can be obtained by modifying the surface of particles with different surfactants; (3) the surface of the particle is covered with a layer (or several layers) of surfactant, which is not easy to coalesce between the particles and has good stability; and (4) the interfacial properties of LNPs can be improved by coating the surface with surfactants. However, this method also has some disadvantages, such as a large amount of emulsifier, a small monomer concentration, and poor purity of product particles.

### 2.5. Spray Freezing Method

Spray freezing is a process in which lignin solution is atomized, frozen into ice particles by contacting with cold medium (such as liquid nitrogen and cold air flow), and then the frozen particles are dehydrated and dried to obtain nanoparticles [35].

Mishra et al. reported a simple method for synthesis of LNPs by spray freezing [36]. Lignin can be well dissolved in dimethyl sulfoxide (DMSO), and DMSO itself has a high melting point. Based on the above two points, the authors dissolved lignin in DMSO and sprayed the resulting solution onto copper plates cooled by liquid nitrogen using a hand-held sprayer. Because of DMSO’s high melting point, when the droplets hit the copper plate they immediately freeze and form particles. This method can avoid the interaction among lignin, solvent, and non-solvent, and the good solubility of lignin in DMSO is conducive to the stability of LNPs.

The preparation of LNPs by spray freezing method has the advantages of continuous production and simple operation, but the particle yield is low and the particle uniformity is poor.

The preparation details and properties of LNPs by different methods are shown in Table 1.

## 3. High Value Utilization of Lignin Nanoparticles

### 3.1. UV Protection

The most widely used sunscreen active ingredients on the market, whether natural or synthetic, are small organic molecules. They are usually insoluble in water, have the potential to penetrate the skin, and can cause skin allergy symptoms after long-term use. It is obviously beneficial to discover natural polymer anti-ultraviolet active substances and develop polymer sunscreen. Lignin contains a large number of phenols, ketones, and intramolecular hydrogen bonds, which has great potential in ultraviolet resistance. In addition, lignin from various sources has been shown to be safe and lignin-based capsules are not cytotoxic [43].

Qian et al. prepared LNPs by self-assembly method, in which sizes and structures are different, and then mixed it with pure skin cream to develop lignin-based sunscreen [44]. The result shows that the emulsion with lignin nanoparticles as the active ingredient has better sunscreen performance. The sun protection factor (SPF) of the sunscreen decreases with the increase of the particle size of LNPs.

**Figure 6:**
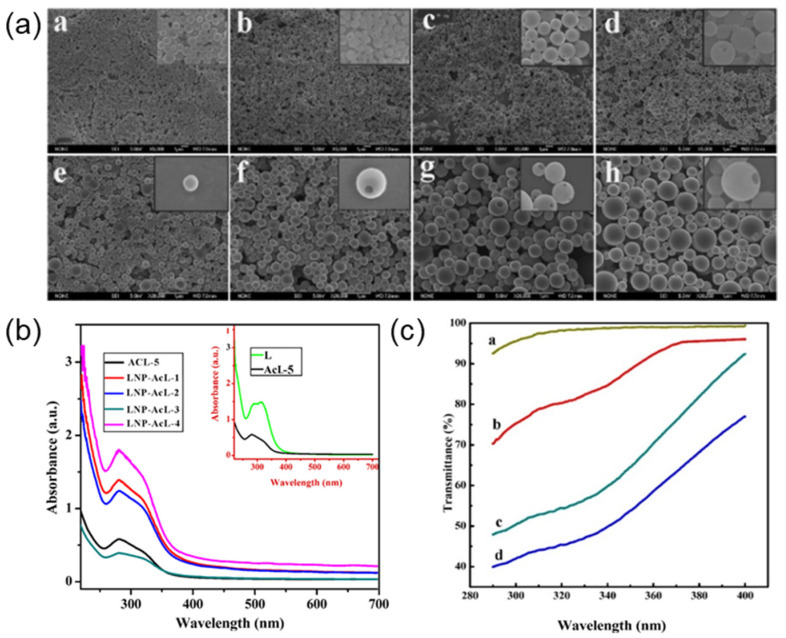
(**a**) SEM of prepared LNPs; (**b**) UV spectrum of ACL and LNP-AcL; (**c**) UV- resistance effect of LNP and LNP-AcL. Reprinted with permission from Ref. [12]. Copyright 2018 American Chemical Society.

Wang et al. prepared different kinds of lignin sunscreens by mixing LNPs with pure creams [12]. The ultraviolet (UV) transmittance is shown in Figure 6. The result shows that the SPF value of pure cream is 1.03, while the SPF value of cream containing different LNPs is between 1.26 and 2.23. The SPF increase of lignin sunscreen is mainly related to the size of LNPs. The author thinks that the good sunscreen performance of LNPs may be due to the conjugation of lignin during the preparation of nanoparticles. Methoxy groups in S- and G-type lignin play an important role in the conjugation of lignin. The π–π accumulation between the aromatic rings in the sunscreen and lignin nanoparticles also contributes to the improvement of sunscreen performance.

Trevisan et al. isolated pure lignin from elephant grass and prepared LNPs and lignin acetate nanoparticles (ACLNP) by self-assembly method using pure lignin as raw material [37]. Due to the good biocompatibility and UV resistance properties of lignin, the authors added LNPs to neutral creams and successfully formed tinted sunscreens with UV protection properties. The creams containing LNPs (3.0 and 10 wt%) and ACLNP (10 wt%) showed lower transmission in the visible region compared to commercial sunscreens with SPF 30. Among them, the creams containing 10 wt% ACLNP had the lowest transmittance in the visible region. Furthermore, compared with the antioxidants on the market, the LNPs prepared by authors have higher antioxidant activity.

Lee et al. extracted light-colored lignin (CEL) from rice husk and prepared spherical nanoparticles of CEL (CEL-NP) which was combined solvent transfer and ultrasonic [45]. The authors added CEL and CEL-NP to commercial moisturizers, and studied the UV protection performance of the mixed cream. The results indicated that the lignin-containing cream showed obviously lower transmittance in the range of UVA and UVB comparing to the single moisturizer. Notably, the UV transmittance of the emulsion with CEL-NP was significantly reduced. In addition, the authors found that CEL-NP had a synergistic effect with organic sunscreens, with an overall increase of approximately 5-fold in SPF and UVA PF values for organic sunscreens containing 5 wt% CEL-NP, while there was little synergy between CEL-NP and inorganic sunscreens

### 3.2. Antibacterial Agent

Phenolic compounds in lignin play an important role in its antibacterial properties, especially its side chain structure and functional groups. In general, phenols have double bonds at the α and β positions of the side chain, and methyl groups at the γ position, giving them the strongest antibacterial properties [46].

Richter et al. embedded silver ions into LNPs and then coated polydiallyl dimethyl ammonium chloride to form a biodegradable substitute for Ag nanoparticles [47]. PDAC coating can promote the adhesion of microbial cell membranes and has some antibacterial activity of its own. However, controls of lignin nanoparticles without PDAC or silver ion loading showed lower antibiotic efficiency, which suggests a synergistic effect of these drugs.

**Figure 7:**
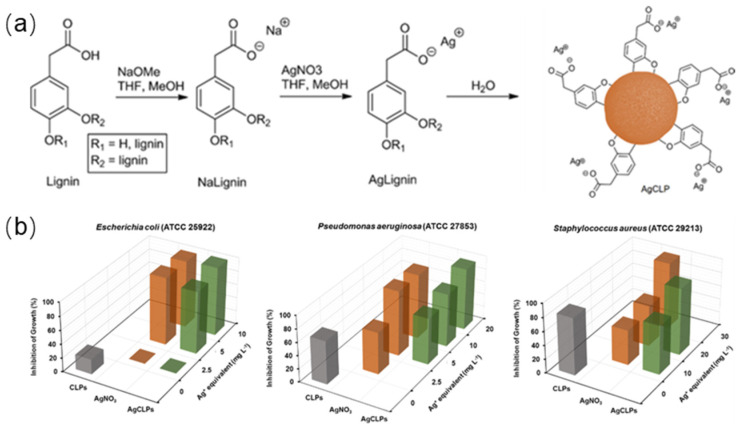
(**a**) Simplified Scheme of AgCLP formation; (**b**) quantification of inhibition of growth as a function of Ag^+^ equivalent of AgCLPs and control samples on CLPs and AgNO_3_ after 24 h exposure. Reprinted with permission from Ref. [48]. Copyright 2019 American Chemical Society.

Lintinen et al. prepared colloidal silver carboxylate lignin particles (AgCLPs) by deprotonation of an anhydrous wood organic solution, followed by ion exchange with silver nitrate, and solvent exchange to form colloids [48]. Silver will not be released from the particles in deionized water, but can be released under physiologically acidic conditions, manifested by low silver load and good antibacterial effect (Figure 7).

Maldonado-Carmona et al. prepared acetylated lignin water-dispersible nanoparticles (THPP@AcLi) by adding 5,10,15,20-tetrakis(4-hydroxyphenyl)-21H,23H-porphyrin (THPP) to acetylated lignin solutions of acetone [49]. THPP@AcLi was characterized by TEM, DLS, and Zeta potential, and it was found that THPP@AcLi retained the photosensitive activity of porphyrin. Compared with acetylated lignin nanoparticles (@AcLi), THPP@AcLi can generate singlet oxygen more efficiently. In addition, two Gram-negative bacteria (*Escherichia coli* and *Pseudomonas aeruginosa*) and three Gram-positive bacteria (*Staphylococcus aureus*, *Staphylococcus epidermidis*, and *Enterococcus faecalis*) were used to investigate the antimicrobial activity of THPP@AcLi. The results showed that THPP@AcLi reduced the survival rate of Gram-positive bacteria to 0.1% when exposed to low dose white LED (4.16 J/cm^2^), but did not reduce the survival rate of Gram-negative bacteria. The author’s further research aims to reduce the survival rate of Gram-negative bacteria, expand the application range of @AcLi, and apply THPP@AcLi to water pollution control and other aspects.

### 3.3. Nano Filler

LNPs have been used in many fields. Compared with lignin, nanoscale lignin particles have higher glass state transition temperature, melting temperature, and crystallization temperature, and better thermal stability. Therefore, they can be efficiently used as natural fillers of nanocomposites [50]. In rubber materials, compared with carbon black, lignin has the advantages of low density, non-conductivity, and light color, so it can replace carbon black to make rubber products with light color. If the lignin powder is directly mixed with rubber, the interaction of hydrogen bonds between lignin molecules in the mixing process will make the lignin bond, and it is difficult to disperse in the rubber [51]. Eventually, the strength of the rubber is difficult to change.

Jiang et al. slowly added the lignin sulfate solution at pH 12 into the rapidly stirred polydiallylammonium chloride solution, formed a lignin-polydiallylammonium chloride complex with an average particle size of less than 100 nm by self-assembly method [52]. Then, the colloidal particles prepared were compounded with rubber to prepare nanocomposites. It is found that the lignin nanoparticles can be evenly dispersed in rubber, which is beneficial to accelerate the hardening of rubber and enhance the thermal stability and mechanical properties of rubber–lignin nanocomposites.

Wang et al. filled the prepared lignin nanoparticles into gluten to prepare nanocomposite membrane materials. After adding LNPs, although the transparency of the nanocomposite membrane material will decrease, the presence of LNPs will increase the ultraviolet absorption capacity of the nanocomposite membrane material [53]. At the same time, LNPs enhance the thermal stability and mechanical properties of the membrane, but weaken the hydrophilicity, which is beneficial to expanding the application of the gluten substrate.

Yang et al. mixed LNPs with polylactic acid, and prepared polylactic acid films by solvent extrusion and solvent casting [54]. The result shows that the LNPs cannot disperse evenly in the polymer matrix for polylactic acid thin films prepared by solvent casting method. For polylactic acid thin films prepared by solvent extrusion method, LNPs can be uniformly dispersed in the polymer matrix. When the mass fraction of LNPs reaches 1%, the tensile strength, modulus, and elongation at break of polylactic acid thin films are significantly higher than that of films without LNPs.

Bian et al. used cellulose nanofibrils (CNF), polyvinyl alcohol (PVA), borax and LNPs as raw materials to prepare a hyperelastic composite hydrogel [55]. It was found that the concentration of free LNPs plays a key role in the viscoelasticity of the composite hydrogel. LNPs were used as nano-spacers to fill the 3D network, which enhanced the interactions between the polymers and improved the viscoelasticity and thermal stability of the hydrogel.

### 3.4. Drug Transport Carrier

LNPs are characterized by low cytotoxicity and biocompatibility, so they can be applied to tissue engineering, bio-based pharmaceutical carriers and drug sustained release, and also have the function of repairing, replacing, and enhancing specific tissues or organs [56].

**Figure 8:**
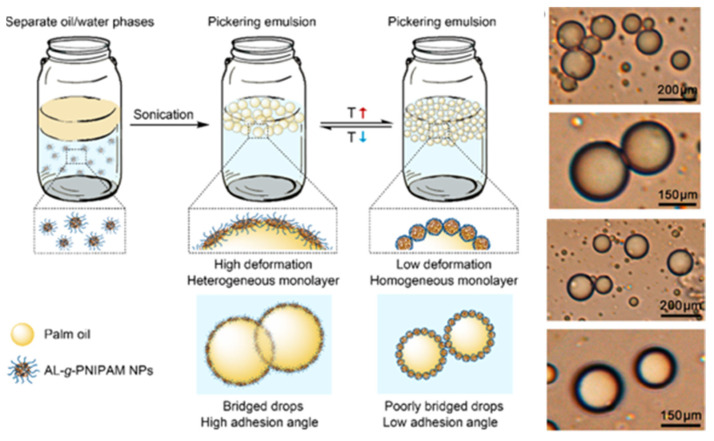
AL-*g*-PNIPAM NPs stabilized palm oil emulsions for heat-controlled release of photosensitive and low water-soluble drugs. Reprinted with permission from Ref. [57]. Copyright 2019 American Chemical Society.

Dai et al. used industrial waste lignin to graft poly-N-isopropyl acrylamide onto lignin by atomic transfer radical polymerization, prepared a thermally responsive lignin copolymer, and then formed self-assembling nanoparticles [57]. The nanoparticles can improve the stability of palm oil emulsions containing trans-resveratrol, making it useful for the storage and heat-controlled release of photosensitive and low water-soluble drugs (Figure 8).

Li et al. prepared pH-responsive lignin-based composite micelles in green solvent using purified alkali lignin as raw material [58]. TEM images showed that the composite micelles were uniform spherical nanoparticles. It was found that more than 74.44% of the drugs could be encapsulated by hydrophobic interaction when ibuprofen was used as the drug loading model. In vitro release behavior is pH-dependent and controllable. This study provides a new method for the preparation of oral lignin delivery carrier, which is of great significance for the high-value utilization of lignin.

Mendez et al. synthesized LNPs from lignin-corn-poly (lactic-co-glycolic) acid by emulsion evaporation. The effects of LNPs on the transport of MFZ in soybean hydroponics were studied using methoxyfenozide (MFZ) as a model [59]. The result showed that LNPs were able to significantly promote the transport of non-systemic MFZ from soybean roots to aerial tissues within 24 h. This study provides a new way to improve the accuracy of pesticide delivery, which may provide an efficient aid for sustainable nano-agriculture.

**Figure 9:**
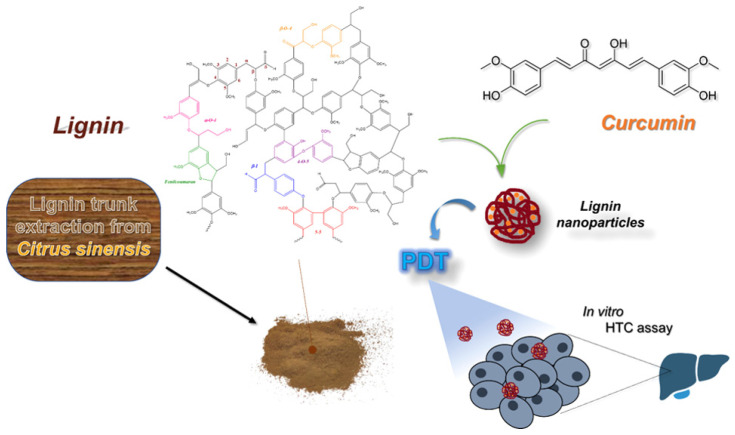
Illustrations of the preparation procedures of LNPs and application of cancer therapy. Reprinted with permission from Ref. [60]. Copyright 2021 American Chemical Society.

Porto et al. extracted lignin from orange trunk by alkaline pulping process and synthesized LNPs by solvent transfer method using this lignin [60]. Nanocurcumin capsules were prepared by encapsulating curcumin (1%, 5%) in LNPs, and the encapsulation rates of curcumin in both concentrations were above 90% (Figure 9). In order to study the potential of nano-curcumin capsules in biomedicine, photodynamic therapy experiment was carried out. The result showed that LNPs with 5% curcumin had selective toxicity to cancer cells.

Alqahtani et al. prepared LNPs by interfacial polymerization/cross-linking and used them to load curcumin [61]. The physicochemical properties of curcumin nanoparticles were characterized. The average particle size of curcumin nanoparticles was 104 nm, and the encapsulation rate of curcumin in LNPs was 92%. In vitro release experiments, the authors found that the curcumin-loaded LNPs had high stability in simulated gastric juice. Subsequently, they also carried out cell viability studies, and found that compared with free curcumin, curcumin nanoparticles enhanced the cellular uptake and intestinal permeability of curcumin, and increased the apparent permeability of the monolayer of CacO-2 cells by five times. In addition, in vivo pharmacokinetic experiments showed that LNPs loaded with curcumin significantly increased the bioavailability and half-life of curcumin compared with oral curcumin suspension. Furthermore, Alqahtani et al. characterized the wound healing activity of curcumin-loaded LNPs. Cytotoxicity assays showed that curcumin nanoparticles were biocompatible, non-cytotoxic, and did not interfere with cell proliferation during the wound healing process [62]. Curcumin-loaded LNPs exhibited potent antibacterial activity against *Staphylococcus aureus* in vitro. In vivo studies showed that curcumin-loaded LNPs significantly improved wound healing activity compared to blank nanoparticles or curcumin solution. In summary, LNPs can be used as a potential nanocarrier for the delivery of orally administered lipophilic molecules (e.g., curcumin). And curcumin-loaded LNPs are expected to be a delivery system for accelerated wound healing.

In addition to using LNPs to encapsulate drugs, Alqahtani et al. investigated the potential of LNPs as vaccine adjuvants [63]. The authors prepared ovalbumin lignin nanoparticles (OVA-LNPs) by adding ovalbumin (OVA) to lignin solution using solvent evaporation technique. The size of nanoparticles was 216 nm and the encapsulation rate of OVA antigen was 81.6%. In vitro studies showed that LNPs were non-cytotoxic and that a significantly higher percentage of antigen was taken up by dendritic cells encapsulated in LNPs compared to free OVA. Immunological studies in mice showed that IgG antibody titers produced by OVA-LNPs were notably higher than those produced by OVA with free OVA and OVA with alum added. These findings imply that LNPs are a promising vaccine adjuvant and ovalbumin delivery system to induce long-term immune responses.

### 3.5. Adsorbent

As an inexpensive and easy-to-use paper byproduct, lignin has its own adsorption capacity, which can be used for the removal of heavy metal ions, dyes and organic pollutants [64]. Because of the presence of various active functional groups (such as hydroxyl, carbonyl, carboxyl, methyl, etc.), lignin can be chemically modified to obtain lignin-based adsorbent materials with high efficiency and to improve the commercial value of lignin [65].

**Figure 10:**
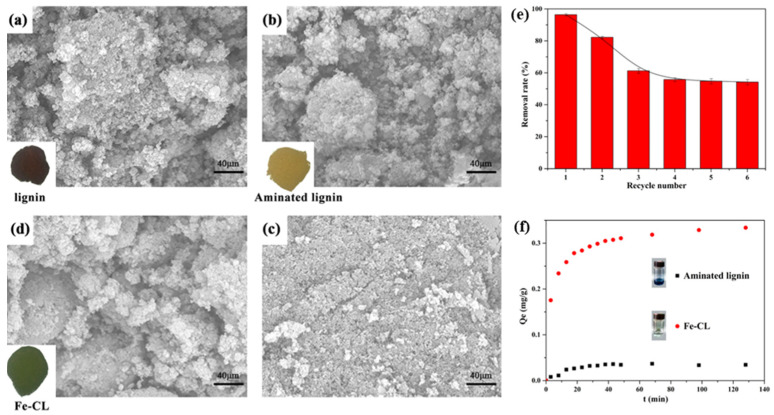
(**a**–**d**) SEM of lignin, aminated lignin, Fe-CL and phosphate-loaded Fe-CL; (**e**) Cycle adsorption of batch experiments for Fe-CL; (**f**) Phosphate adsorption kinetic of aminated lignin and Fe-CL. Reprinted with permission from Ref. [67]. Copyright 2017 American Chemical Society.

Ma et al. synthesized magnetic lignin-based carbon nanoparticles (MLBCN) by precipitation-carbonization method and investigated their adsorption properties on methyl orange [66]. It was shown that the adsorption percentages of methyl orange were much higher in the concentration range of 20–40 mg/L, indicating that the removal of methyl orange by MLBCN was more complete at lower concentrations (20–40 mg/L). In addition, the authors compared MLBCN with other published absorbents and found that the adsorption capacity of MLBCN was significantly superior to other published absorbents. This study shows that MLBCN is an effective green adsorbent for the removal of methyl orange, which is important for the high-value utilization of lignin and resource recovery. Luo et al. modified lignin to aminated lignin by Mannich reaction, and then chelated FeCl_3_ with aminated lignin to prepare Fe (III)-complexed lignin (Fe-Cl) adsorbent [67]. The adsorption experiments and characterization showed that the phosphate adsorption mechanism of Fe (III)-complexed lignin (Fe-Cl) followed the complex mechanism of iron and phosphate on Fe-Cl (Figure 10). Moreover, the adsorption experiment further confirmed the strong interaction between phosphate and Fe-Cl, which can effectively remove phosphate. This study demonstrates that lignin is a potential adsorbent that can be used for the removal of low concentrations of sulfate from wastewater, with great environmental and economic benefits in wastewater treatment.

Behboudi et al. synthesized lignosulfonate nanoparticles (LS-NPs) by solvent transfer method using ethanol as the anti-solvent [68]. The authors investigated the adsorption potential of LS-NPs on dye molecules using Safranin-O as a simulated contaminant. The results showed that the maximum adsorption capacity of Safranin-O was 85.14 mg/gr at pH 10. At this time, there was a strong electrostatic interaction between LS-NPs and cationic Safranin-O molecules, so it has a strong adsorption capacity.

Araújo et al. prepared lignin/Fe_3_O_4_ nanoparticles from green coconut fiber using an anti-solvent self-assembly method [69]. The adsorption properties of lignin/Fe_3_O_4_ nanoparticles for textile dyes (methylene blue (MB), cycloparon blue (CB), and rimazole red (RR)) were investigated. Compared with other dyes, MB dye has a higher affinity for Lignin Fe_3_O_4_ nanoparticles. When the concentration of lignin nanoparticles was 40.0 μg/L, the removal rate of MB dye could reach 87.22% of the initial content. The adsorption kinetics study showed that the lignin/Fe_3_O_4_ nanoparticles had a lower equilibrium time and higher adsorption capacity for textile dyes compared with other adsorbents. However, with the increase of reuse times, the adsorption capacity of lignin/Fe_3_O_4_ nanoparticles for dyes reduced sharply. In the future, this irreversible adsorption mechanism can be further explored to improve the performance of lignin/Fe_3_O_4_ nanoparticles in wastewater treatment.

## 4. Summary and Outlook

Due to the complex structure of lignin, its application is greatly limited. The development of LNPs opens up a new direction for the transformation and application of lignin. Compared with traditional inorganic nanomaterials, LNPs have the characteristics of biodegradability, good biocompatibility, anti-ultraviolet, antibacterial, etc., showing great application potential in materials, chemicals, microorganisms, medicine, and other fields. This review focuses on the preparation techniques of LNPs by solvent exchange, mechanical, enzymatic, and interfacial polymerization/crosslinking methods, as well as the application of LNPs in UV protection, antibacterial, nanofiller, etc., providing more ideas for the high-value utilization of LNPs.

At present, the preparation and application of LNPs is gradually accelerating, but is still in its infancy. The prospects and challenges for LNPs in the future mainly include the following aspects: (1) develop efficient separation and purification technology of lignin, which is the key to control the structure and properties of lignin. The structural and functional properties of lignin determine its applications. Lignin and its derivatives have a wide range of functionalities and can be used in various fields. However, all these applications depend on the improvements and innovations of lignin separation, which can provide a stable and continuous source of high purity lignin source for enterprise; (2) develop green, stable and controllable preparation technology of LNPs, which is the premise of large-scale industrial application of lignin. The problems for the current large-scale preparation of LNPs are the complex process, overuse of toxic organic solvents, and uncontrollable LNPs size. In view of these problems, efficient separation technology (such as fractional purification) and preparation methods (such as electrostatic spinning) can be utilized; and (3) broaden the field of high-value utilization of LNPs, especially in biomedicine, intelligent manufacturing and other high-value fields.

## Figures and Tables

**Table 1:** Preparing LNPs by different methods.

Lignin Source	Technique	Reaction Conditions				Size (nm)	Morphology	Yield	Advantages	Ref.
		Solvent	Temp. (°C)	Time	pH					
Elephant grass; acid-alkali extraction; pure lignin	Self-assembly method	Acetone and deionized water	NR	10 min	NR	55 ± 26; 86 ± 29	Spherical; hollow nanospheres	37 ± 3% (LNP); 36 ± 3% (AcLNP)	Simple and greener method	[37]
Corncob alkali lignin; mixed softwood kraft pulping; Kraft lignin	Self-assembly method	Deep eutectic solvents, H_2_SO_4_, NaOH and deionized water	25	60 min	4, 5, 6	30.4–138.2	Spherical	90.3%	Eco-friendly, high yield	[38]
Corn cob; alkali lignin	Self-assembly method	Methanol, ethanol, and Tetrahydrofuran (THF)	NR	NR	NR	130	Spherical	NR	Certain stability and excellent biocompatibility	[14]
Wheat straw	Self-assembly method	Aqueous p-toluene sulfonic acid and deionized water	NR	10 min	NR	295	Oblate spheroidal	81%	Facile and green method	[39]
Corncob residue; enzymatic mild acidolysis	Dialysis method	Dimethyl sulfoxide, sodium acetate buffer solution	NR	At least 2 days	4.7	60–200	Spherical	NR	Controllable and larger size range	[40]
Corn stover; Tailored lignin (SOFA)	Dialysis method	THF and deionized water	120	15 min	1.8, 3.8, 6.2, 13.0	130	Spherical	NR	High-quality, uniform	[41]
Kraft lignin	Dialysis method	THF and deionized water	NR	At least 24 h	1–11	200–500	Spherical, colloidal nanoparticles	NR	Very stable, scalable method	[18]
Alkali lignin	Acid precipitation method	Ethylene glycol, three acids (HCl, H_2_SO_4_ and H_3_PO_4_).	35	2 h	2.5–4.7	32.8 ± 6.0 (HCl); 58.9 ± 8.6 (H_2_SO_4_); 54.1 ± 6.7(H_3_PO_4_)	Spherical	87.9%, 85.4%, and 78.5% for HCl, H_2_SO_4_, and H_3_PO_4_	Simple method, high yield	[21]
Alkali lignin and hardwood dioxane lignin	Solvent exchange method	Acetone and deionized water	20	10 min	NR	80–104	Spherical	63% (DLNP); 33% (ALNP)	High yield, excellent stability	[42]
Wheat straw lignin, sarkanda grass lignin	Ultrasonication	H_2_O	NR	60 min	NR	100	Spherical	NR	Simple physical method, no organic solvents	[28]
Kraft lignin	Mechanical shearing	H_2_O	NR	1, 2, 4 h	NR	<100	Irregular	NR	Simple mechanical treatment	[26]
Cotton stalk	Enzymatic hydrolysis	H_2_O	Ice bath	1 h	NR	37.3 ± 2.3	Non-uniform spherical	45.3%	Eco-friendly	[31]
Sodium lignosulfonate	Interfacial polymerization/crosslinking	butyl acetate, H_2_O	60	6 h	NR	100–400	Nanocapsules	NR	Particle size is uniform and controllable	[34]
Alkali lignin	Spray freezing	DMSO	4	NR	NR	150	Spherical	NR	Continuous production and simple operation	[36]

NR: not reported.
